# Hirayama Disease in a Young Male: A Case Report

**DOI:** 10.31729/jnma.8804

**Published:** 2024-11-30

**Authors:** Suman Paudel, Prerana Singh Rokaha, Pratik Singh Rokaha, Lalit Karki, Paras Thapa

**Affiliations:** 1 Department of Radiology, Kathmandu Medical College and Teaching Hospital, Sinamangal, Kathmandu, Nepal; 2 Kathmandu Medical College and Teaching Hospital, Sinamangal, Kathmandu, Nepal; 3 Lumbini Medical College and Teaching Hospital, Tansen, Palpa, Nepal

**Keywords:** *amyotrophy*, *magnetic resonance imaging*, *monomelic*, *juvenile spinal muscular atrophies*

## Abstract

Hirayama disease is a rare benign neurological disease that affects the anterior horn of the spinal cord at C5 to T1, mainly at C7 and C8 due to imbalance growth between the vertebral column and the dura mater leading to microcirculatory disturbances in the anterior portion of the spinal cord due to overstretched cord, especially during flexion of the neck causing unilateral or rarely asymmetrically bilateral upper limb weakness and muscle wasting.

It is a case of a 21-year-old boy presented with weaknesses in his left hand and forearm for 2 years which aggravates during cold weather and flexion of the neck. A plain x-ray of the neck and MRI of the cervical spine were conducted which show the features of Hirayama disease.

## INTRODUCTION

Hirayama disease, also known as monomelic amyotrophy, juvenile amyotrophy of the distal upper extremity, and juvenile muscular atrophy of the distal upper extremities.^1, 2^ It is a rare disease with only 1500 case studies noted in the literature and the maximum of those studies are from Japan (333 cases), then India (279 cases), and then China (179 cases).^2^ The majority of these cases occur in young males between the ages of 15 and 25 in Asian countries like Japan and India.^1,2^

Here we present the case of a 21-year-old boy with Hirayama disease involving the left hand and forearm for 2 years.

## CASE REPORT

21-year-old boy with a history of slowly progressive weakness of the left hand and forearm for 2 years presented to neurology OPD. Weakness was aggravated during cold weather. Also, he complained of aggravation of his weakness during the flexion movement of the neck. He also noticed gradual atrophy of the muscle of the left forearm and hand during this period. There was no history of pain, abnormal sensation, diplopia, dysphagia, ptosis, muscle cramps, fasciculations, headache, or neck pain. There was no significant past medical/surgical/family history.

On clinical examination, there was evidence of atrophic changes involving the thenar, hypothenar, and interosseous muscles of the left hand and the muscles of the left forearm. Brachioradialis was spared. Deep tendon reflexes were normal and symmetrical on both sides. Sensation to pin-prick, vibration, and joint position was intact.

**Figure 1:**
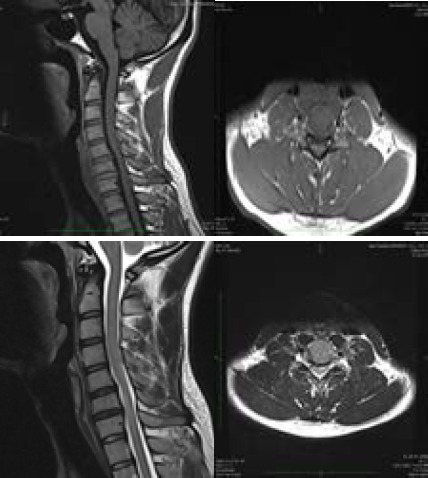
T1 and T2 sagittal and axial images show atrophy of the spinal cord at C5 to C7 vertebral level.

**Figure 2:**
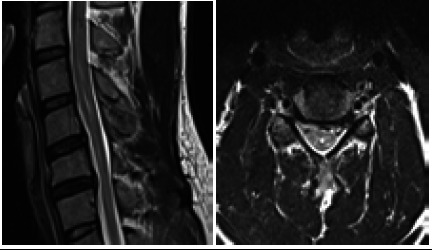
T2 weighted sagittal and axial images in neural position show the presence of the dilatation of the central canal at C4 to C6 vertebral level representing syrinx.

**Figure 3:**
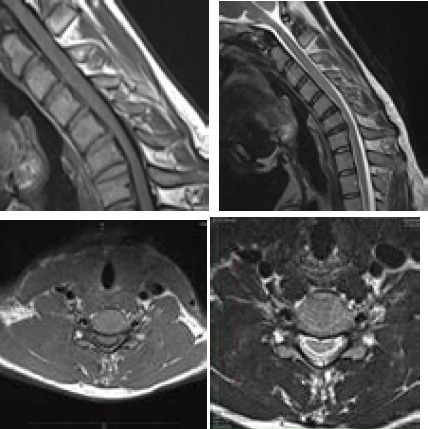
Sagittal and axial T1/T2 weighted images on flexion position showing anterior displacement of the posterior wall of the cervical dural canal at C5-C7 vertebral level causing flattening of the spinal cord at the same level. Also, note the presence of the crescent-shaped T1/T2 intermediate signal intensity area in the posterior epidural space.

**Figure 4:**
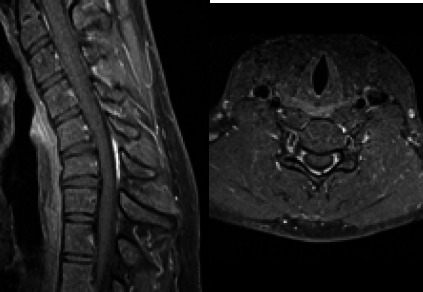
Sagittal and axial T1 weighted fat saturated post-contrast study on flexion position showing avid enhancement of the posterior epidural space at C5-C7 vertebral level.

The plain cervical spine radiographs showed no definite abnormality. Multiplanar multisequence magnetic resonance imaging (MRI) of the cervical spine in neutral and flexion position was performed with a Siemens 3T scanner. There was evidence of spinal cord atrophy at C5 to C7 vertebral level (Figure 1). Also, there was presence of the dilatation of the central canal at the level of C4 to C6 representing syrinx (Figure 2). Similarly, MR images on flexion position showed anterior displacement of the posterior wall of the cervical dural canal at the level of C5-C7 causing flattening of the spinal cord at the same level (Figure 3). Crescent shaped area showing T1/T2 intermediate signal intensity is seen along the posterior epidural space at the level of C5-C7, which on T1 weighted fat saturated post-contrast study, showed homogeneous contrast enhancement (Figure 4). It disappeared after the patient returned to a neutral position.

Based on the clinical presentation and MR imaging findings, the diagnosis of Hirayama disease was made and the patient was managed conservatively with a neck collar. There was no further progression of symptoms at the 3-month follow-up study.

## DISCUSSION

Hirayama disease is a rare and benign form of neurological condition characterized by focal amyotrophy of the lower cervical cord from C5 to T1 particularly at C7 and C8 myotomes which present with insidious onset and slow progression of unilateral or rarely asymmetrically bilateral weakness which increases on exposure to cold and muscle wasting of upper limb.^3,4^ It is a self-limiting disease mostly seen in young males of 15-25 years of age usually in Asian countries like Japan, India, China, Sri Lanka, Singapore, Taiwan and Hong Kong.^1, 2 3^ Usually it is unilateral disease involving the upper limb only, and rarely it is bilateral, and even rarely it presents as lower ipsilateral limb involvement.^5^ Within 3 years of onset of the disease, 70% of the patients see the disease progression and after 5 years in 95% of the patients the progression of disease arrest.^3^ No sensory pathway, cranial nerves, or brain function involvement was seen.^6^

The pathogenesis of the disease is unknown-but it is believed to be caused due to imbalance growth of the vertebral column and the spinal canal contents due to which "tight dural sac" or "overstretch of the cord" in normal position and the forward displacement of the posterior lower cervical dural wall in flexed neck position which causes asymmetrical flattening of lower cervical. ^2 7^ On average, the change in the length between the top of the atlas and T1 at the anterior wall and posterior wall during extension and flexion of the neck is 1.5cm and 5cm respectively.^8^ In normal people when the neck is in extension, the cervical spine slacks and forms transverse folds, and when flexed the dura becomes tighter as the length of the neck increases but is compensated by transverse folds hence forward displacement of the posterior wall doesn't occur.^8^ But in Hirayama there is forward shifting of the posterior dural wall thus causing injury due to chronic compression of blood vessels especially in the anterior horn of the spinal cord from C5 to T1 as the anterior horn is the most sensitive area to ischemic.^4^

Cervical spine MRI with more than 25 degrees neck flexion is done for the diagnosis.^7,9^ The MRI changes described by Hung and Chan are asymmetrical atrophy of the cord in a neutral position and loss of attachment of the posterior sac to the lamina.^10^ Crescent-shaped structures and distorted cervical curvature can also be appreciated in MRI, suggesting Hirayama disease.^7^ Plain X-ray of the neck if done in this disease shows no specific features and only scoliosis can be seen, as it was in our case.^7^

In this case MRI showed features consistent with Hirayama disease.

Tashiro et al. recently outlined the following important criteria for the diagnosis of Hirayama disease to make the challenging diagnosis easy.^3^

1. Distal predominant muscle weakness and atrophy in forearm and hand;
2. Involvement of the unilateral upper extremity in most cases;
3. Onset between the ages of 10 to early 20s;
4. Insidious onset with gradual progression for the first several years, followed by stabilization;
5. No lower extremity involvement;
6. No sensory disturbance and tendon reflex abnormalities;
7. Exclusion of other diseases^3^

All the seven Tashior et al. criteria for the Hirayama disease were fulfilled in our case.

Hirayama disease is a benign self-limiting disease as the progression of the disease continues only for one or two years and eventually, progression stops abruptly.^2^ The main line of treatment is to decrease the progression of the disease before it reaches its plateau phase which is done by immobilizing the neck or limiting the flexion movement of the neck by using a cervical collar.^1,2^
